# Racial Bias in the US Opioid Epidemic: A Review of the History of Systemic Bias and Implications for Care

**DOI:** 10.7759/cureus.3733

**Published:** 2018-12-14

**Authors:** Taylor N Santoro, Jonathan D Santoro

**Affiliations:** 1 Miscellaneous, Columbia University, New York, USA; 2 Neurology, Massachusetts General Hospital, Boston, USA

**Keywords:** minority, african-american, race, opioid epidemic

## Abstract

The opioid epidemic has been declared a US national public health emergency. Discrepancies in the rates of abuse and access to treatment exist among non-white minorities. A narrative literature review evaluated the minority racial disparities in opioid use, abuse, and care in the US. Racial disparities in the prescription of opioid-containing compounds are dramatic with the non-white individuals being prescribed at half the rate. Historical and cognitive biases may have insulated the non-white minorities, while the minorities have limited access to treatment. Physician bias, media portrayal of opioid abuse disorders, and governmental regulation are a polyfactorial root of racial inequity in the opioid epidemic. As part of the national response, addressing these issues will be an important factor in curbing this epidemic.

## Introduction and background

Pain management is a significant problem in American healthcare. In the United States, chronic pain accounts for up to 20% of all physician office visits, while acute pain is the most common cause of emergency room visits [1]. The growing burdens of pain management were originally recognized in the 1990s when the Joint Commission on Accreditation of Health Care Organizations (JCAHO) identified a need to make pain “visible” and promoted new national standards wherein healthcare workers would have the proper tools to improve the quality of pain control for patients [2]. In 1996, in the setting of a national push for the identification of pain as a primary medical disorder, oxycodone hydrochloride, commonly known as OxyContin™, was approved by the Food and Drug Administration (FDA) as a “minimally addictive pain reliever” [3]. At the time, this compound provided physicians a significant upgrade in their arsenal of pain management tools with superficially minimal risks to patients as reported by their producers.

While oxycodone and similar compounds had an impressionable effect on pain relief and management of patients, there was also a rise in prescription painkiller use and abuse in the United States following the introduction of these agents [4]. The Center for Disease Control and Prevention (CDC) categorized the rise in opioid use into three main “waves”: the first wave starting in the late 1990s, which was attributed to a rise in prescription opioids for both acute and chronic pain management; the second wave starting in 2010, attributed to increasing heroin use, often in conjunction with other synthetic opioid compounds; and the most recent and deadly wave starting in 2013, attributed to the increasing access to and the utilization of high-concentration synthetic opioids (e.g. illicitly-manufactured fentanyl) [5].

The magnitude of this problem is most evident in the rise of opioid-related overdoses and deaths in the United States. In 2016, 64,070 Americans died from drug overdoses with 66% of those deaths involving opioid compounds [4]. Analyzing this statistic further, the rate of Caucasians who died from an opioid-related overdose was 79%, but only 10% for the non-white minorities, identifying an alarmingly discrepant racial profile of opioid users in the United States [5].

The cause of the opioid epidemic in the United States cannot be traced to one particular source; rather the epidemic evolved to its current state from many concurrent issues in the societal perspective of drug users and healthcare practices and policies. These complex and interconnected factors also resonate discrepant racial trends of opioid users and the currently available treatments for opioid addiction. Physician practice, along with other systemic problems, has created disparity in the race/ethnicity of prescription opioid users and more gravely in opioid-related overdoses and deaths. This inequality begins with pain management practices and extends to the treatment responses and the availability of addiction resources. This narrative literature review seeks to address the historical, racial, and cultural biases in the opioid epidemic and how this has shaped the medical and governmental interventions in this growing epidemic.

## Review

Moral and neurobiologic etiologies of disease

Historically, addiction was perceived as a moral failing of the individual, with the identification that persons acted voluntarily in the search of and continued use of drugs and that personality flaws were the explanation for the inability to cease use [6-7]. Although this viewpoint has largely been abandoned in the medical community, the identification of addiction as a “moral” condition has persisted as a public viewpoint with many Americans holding negative views of individuals who have substance use and abuse disorders [8]. Although strong data exists for a neurobiologic basis for the lack of control in substance use and abuse disorders, the contextualization of these medical problems as moral flaws has undoubtedly made seeking access for care stigmatized, and thus, underutilized [9].

In the 1990s, the National Institute of Drug Abuse’s (NIDA) neuroscience program redefined the framework of addiction by identifying a neurobiological component that warranted study. The NIDA pushed for scientific programming that addressed a brain disease model in substance use and abuse disorders, endeavoring to identify the neurologic substrates of behavior [3]. This change in disease modeling and shift towards identifying the physiologic basis for the disease may have also had the dual effect of changing the conceptualization of addiction from being a reflection of morality to altered brain physiology. This perspective was intended to reduce the stigma associated with substance use and abuse disorders as it broke the association with morality, instead refocusing the central problem on biochemical aberrations of the individual [3,9]. A shift to the utilization of a neurobiologic model for substance use and abuse disorders provided a step towards medicalization of these disorders, although inconsistencies in the model were present.

While the initial conceptualization of a solely neurobiologic model of substance use and abuse disorders was idealistic, it did not address the interplay between environmental and physical triggers of the disease. Although later research identified complex biochemical and epigenetic variables can affect addiction [10-11], the early models did not have access to this data for the explanation of weakness in the strictly neurobiologic model.

The incorporation of sociologic, public health and basic science principles into an integrated approach to substance use and abuse has occurred over time. The identification of risk factors that play into drug use and can lead individuals to a greater likelihood of substance use and abuse as well as the biological basis for these factors implore a multidisciplinary perspective to understand this disease that includes the neurobiologic model for addiction [10]. The variable of race as an independent risk factor for substance use and abuse has long been intertwined in the history of addiction in the United States and continues to pose a problem to the responses to addiction. This is particularly true for the individual and broad responses to the opioid epidemic.

Biases and stereotyping of addiction in healthcare practices

It is well noted that the non-white minorities are more likely to be undertreated in a variety of medical settings and specifically receive inadequate treatment for pain conditions [1,12-13]. Interestingly, this may not be only a medical system bias as a recent study assessing the opinions of white laypersons, medical students, and medical residents identified a continued belief that the black body is biologically “different” from the whites and actually “stronger” [14]. These beliefs may stem from the days of slavery in the United States where scientists and physicians used pseudo-scientific studies on slaves to justify the need for slavery, often citing biological differences between white and non-white persons [14]. On an individual level, these biases may be difficult to consciously identify; however, the continued application of these beliefs can be detrimental to the current and future clinical and therapeutic interventions of the medical practitioner [15].

Misinformed belief systems and maladaptive cognitive schema like these can have tangible effects on the quality of care, especially in the pain management by a provider because of a potential unconscious bias regarding “strength” or “frailty” in a patient of a particular race or an ethnic group [14,16]. There has yet to be empirical data about a genetic difference that would give evidence that there is a real biological difference between different racial groups that would justify the differences in the pain management strategies.

Stereotyping in healthcare continues to persist whether it be through unconscious or “automatic” stereotyping, or goal-modified stereotyping, which is influenced by conscious thought [12,15-16]. These stereotyping mechanisms are more prevalent in situations where there is a “cognitive overload”; such as stressful work environments or situations of limited access to adequate information to make informed decisions. Situations such as this may occur during time-limited appointments with physicians, especially given the complex nature of both acute and chronic pain disorders. This mechanism of stereotyping uses subjective reasoning and personally held beliefs that can facilitate the decision-making process by “bridging the gap” when information or time is insufficient to make a well- informed decision [12]. To overcome this issue, medical providers must be aware of the particular situations when stereotyping is more prevalent, when cognitive resources are limited, and additionally receive the proper skills and support to avoid making inappropriate decisions that may have detrimental effects on the quality of care for marginalized populations.

Historical criminalization of illicit drugs and effects on opioid administration and care

Throughout American history, race, ethnicity, and class have influenced the public’s opinion of drug use and addiction. This was observed most recently on a large scale during the crack cocaine epidemic of the 1980s in the United States. During this period, the political campaign, known as the “War on Drug”, was used as a response to counteract the increasing rates of use and abuse of this compound. Media representations of minorities (specifically African-Americans and Latinos) in urban inner-cities were frequently depicted as addicts and criminals, while the Whites were portrayed by the media as “victims” [17-18]. The push for incarceration as a means to handle the increasing prevalence of crack cocaine use and abuse was realized with the 1986 Anti-Drug Abuse Act signed by the President Ronal Reagan, which imposed a minimum sentencing law for the distribution of crack cocaine and powder cocaine. This law included a 100:1 ratio between crack cocaine versus powder cocaine distribution, where crack cocaine distributors were more harshly penalized [19]. In 1988, the Omnibus Anti-Drug Abuse Act expanded the law to allow for harsher criminalization of the crack cocaine users. A possession of 5 g would result in a five-year minimum sentence, while powder cocaine possession for the same amount would only result in a one-year maximum sentence [20].

The focus for this government response was on the individual users, abusers, and the distributors of the drugs and also distinguished different responses between crack cocaine and powder cocaine irrespective of the fact that both drugs have similar chemical makeups [18,21]. Abusers of the drugs were not offered medical treatment options instead of or as a supplement to incarceration, which intensified the public perspective that these individuals had a moral failing, rather than a disease. The view that addiction was a disease was still years from being linked to addiction with the introduction of the brain disease model in the early 1990s.

Media portrayal of opioid use

The media has a profound effect on the perspectives of the American public. In a study by McGinty et al. [22], participants were presented with specific vignettes that portrayed individuals with specific mental illnesses or substance abuse disorders in different lights: that the individual had gone through a successful course of treatment versus not going through treatment at all. Opinions were more favorable when the vignette depicted an individual as someone who had successfully completed treatment for their drug use and abuse disorder. There is an effect on the public’s perspective just through the way language and imagery are used in news stories, which can add to misinformed beliefs and stigmas [18]. This phenomenon has created a problem in the opioid epidemic in the United States because of the inconsistency in how the media has portrayed opioid users of different races/ethnicities.

News stories involving the whites and/or middle-class persons with substance use or abuse disorders more frequently include a narrative with clear reasoning to their abuse of opioids that is often attributed to the external factors rather than an inherent moral failing or neurobiologic disorder [18,23-24]. The depiction of persons with substance use and abuse disorders in this manner allows the viewer to empathize with their story while minimizing factors that are not dissimilar with their cognitive schema of substance use and abuse disorders. Conversely, media coverage coming from minority communities were more frequently short where only a name, the arrest, and criminal charges are specified [25]. The lack of a narrative story or the factors associated with use or abuse is published which resonates with the audience and has the potential to add to the stigmas that are already present [8,18,26-27]. The media portrayal of opioid addiction in America has the capability to transform society’s perspective on addiction; therefore, its portrayals must be carefully cultivated to avoid damaging effects that add to stigmatization and discrimination.

Racial inequality in the management of opioid use and abuse disorders

The harmful effect of reinforced biases about persons with substance use and abuse disorders has the potential to affect both public opinion and medical practice. Pain relievers of all varieties are less likely to be prescribed to non-white minorities causing inadequate treatment of pain in non-white individuals [28]. As discussed, there are biases in the practices of medicine for minority populations, and due to the known potential for abuse of opioid medications, healthcare providers are hesitant to prescribe to minority groups.

However, the reasons for prescribing differences among minorities are polyfactorial and influenced by other non-individual factors. When Purdue Pharmaceuticals first released oxycodone hydrochloride to the market in the mid-1990s, their marketing strategy targeted specific providers in the rural and suburban areas (oxycodone hydrochloride was later coined “hillbilly heroin” due to its association with rural populations) [18]. The compound was additionally promoted for the treatment of moderate pain in patients with minimal side effect profiles although the actual data submitted to the FDA did not demonstrate these findings [3]. The timing of the release of oxycodone hydrochloride also seemed to be a significant factor in prescription trends as the association of substance use and abuse in the non-white minority groups was fresh in many providers’ minds following the crack-cocaine epidemic that lasted through the early 1990s in the United States.

A perceived potential for abuse in minorities of these compounds may have made physicians less likely to prescribe these pain relievers to minorities. Interestingly, this may have resulted in an initial insulation of minority communities from use and abuse of prescription opioids [18]. Even with these prescription drugs entering the illegal drug market, the non-medical use of opioid base prescription pain relievers is two times greater in the Caucasians than in the non-white minority [18]. It is possible that these initial prescribing patterns affect the opioid-related overdose and death rates between the whites and non-white minorities observed over the last decade.

Discrepancies in opioid substance use and abuse treatments

Opioid abuse treatment options and availability are affected by inherent biases in the healthcare system. Although public and medical professional attitudes have shifted since the crack cocaine epidemic of the 1980s to a treatment-focused resolution, similar issues regarding care equity remain. One of the most significant obstacles that minority groups face in opioid abuse treatment is limited access to qualified healthcare providers who can assist with pharmacological treatment opportunities and medication-assisted treatments (MATs) [18,28-29]. Moreover, among minorities who do seek treatment through a healthcare provider are more likely to handle treatment with a primary care physician instead of a mental health specialist or addiction specialist and are additionally more likely to be prescribed methadone monotherapy [30-32]. The two main treatment options for opioid dependence, methadone, and buprenorphine are available through very different sectors of the healthcare system with large limitations on which physicians are able to prescribe these compounds.

Methadone has been historically used for opioid abuse and dependence. When methadone was first being studied for its efficacy for treatment, it was used in clinical trials on inner-city minorities for heroin addiction [20,31-32]. There is still an association with the inner-city minority communities and methadone clinics, where they are largely concentrated [3,32]. Methadone is dispensed at clinics, which are tightly regulated by the federal, state, and the local government to ensure healthy practices and limit abuse potential. Treatment is complicated for Medicaid patients due to the inconsistent funding or time-restrictions that are imposed by Medicaid insurance. This is detrimental to the patient as it can impede their success for treatment due to the higher chance of relapse with sub-optimal dosing of methadone [20,32]. Additionally, even when access is available, non-white minorities utilize the services at half the rate of Caucasians due to the financial burden associated with overcoming multiple barriers [29,33]. Further, methadone clinics must be visited on a daily basis to receive proper dosing, which can burden the patient (from both a logistical and economic perspective) and hinder adherence. Non-government funded opioid agonist clinics are more frequently located in areas of high income, further compounding the access to care for minorities who have lower socioeconomic status to Caucasians throughout the country [34]. This is compounded by the fact that opioid recovery can take years and even a lifetime to achieve [35]. A flaw in methadone treatment is the public aspect of the treatment itself, which can deter patients from adhering to programs due to the fear of stigmatization.

In 2000, the Drug Addiction Treatment Act (DATA) allowed physicians to prescribe buprenorphine, or Suboxone®, to persons with opioid substance abuse disorders. This was an innovative pharmacological molecule, due to its properties as an opioid agonist and antagonist that had less abuse potential than methadone [3]. Physicians, at their discretion, can prescribe this compound to patients who then can go to a pharmacy or an OTP to obtain the drug [20]. There are many factors that restrict the access of buprenorphine which include the provider access and payment by insurance. The guidelines within DATA that are enforced by the DEA require physicians to become certified in prescribing buprenorphine, attend the training courses, and also restrict the number of patients that physicians can prescribe the treatment to, most recently only 30 patients during a physician’s first year of certification [3]. These restrictions have the dual effect of limiting the supply of physicians willing to participate in these programs due to the additional barriers for the prescribing privileges, further exacerbating access issues. Additionally, since private practice physicians are more likely to be certified to prescribe buprenorphine, there is less likelihood that minorities have access to these treatments.

The discrepancies in access to care between the non-white minorities and the Caucasians may seem trivial, given the overall lower rates of opioid use in the former. However, in comparing rates of deaths from prescription opioids, non-white minority representation has increased substantially over the past 15 years, especially in the young adult population [36-37]. This has been further compounded by illicit drug users who also abuse opioids, a group where minorities (specifically African-Americans and Latinos) are overrepresented [38-39].

## Conclusions

Inherent, polyfactorial biases in the medical practice of pain management as well as in the opioid abuse treatment are linked to the cause, trends, and difficulty in the resolution of the opioid epidemic in the United States. Although physicians are a large part of the current issue, education alone is not sufficient for this large-scale public health issue. As key players in the treatment of persons with opioid abuse disorders, physicians should also have less regulation in access to prescribing and utilizing medications such as methadone and/or buprenorphine in order to increase access to substance abuse care.
